# Intracellular Sequestration of the NKG2D Ligand MIC B by Species F Adenovirus

**DOI:** 10.3390/v13071289

**Published:** 2021-07-01

**Authors:** Edson R. A. Oliveira, Lenong Li, Marlene Bouvier

**Affiliations:** Department of Microbiology and Immunology, University of Illinois at Chicago, 909 S Wolcott Avenue, Chicago, IL 60612, USA; edsonrao@gmail.com (E.R.A.O.); Lenong@uic.edu (L.L.)

**Keywords:** adenoviruses, adenovirus species F, viral tropism, gut immune system, enteric viruses, immune evasion, NK cells, MIC A and MIC B

## Abstract

The enteric human adenoviruses of species F (HAdVs-F), which comprise HAdV-F40 and HAdV-F41, are significant pathogens that cause acute gastroenteritis in children worldwide. The early transcription unit 3 (E3) of HAdVs-F is markedly different from that of all other HAdV species. To date, the E3 proteins unique to HAdVs-F have not been characterized and the mechanism by which HAdVs-F evade immune defenses in the gastrointestinal (GI) tract is poorly understood. Here, we show that HAdV-F41 infection of human intestinal HCT116 cells upregulated the expression of MHC class I-related chain A (MIC A) and MIC B relative to uninfected cells. Our results also showed that, for MIC B, this response did not however result in a significant increase of MIC B on the cell surface. Instead, MIC B was largely sequestered intracellularly. Thus, although HAdV-F41 infection of HCT116 cells upregulated MIC B expression, the ligand remained inside infected cells. A similar observation could not be made for MIC A in these cells. Our preliminary findings represent a novel function of HAdVs-F that may enable these viruses to evade immune surveillance by natural killer (NK) cells in the infected gut, thereby paving the way for the future investigation of their unique E3 proteins.

## 1. Introduction

**Figure 1:**
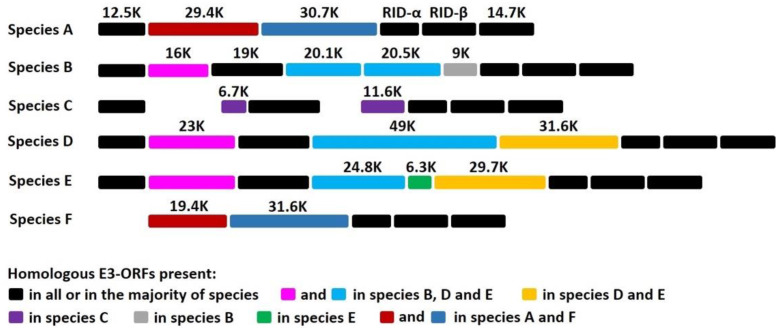
Sequence alignment showing the coding potential of E3 regions of the most common HAdVs-A, -B, -C, -D, -E, and -F. The expected molecular mass of each gene product is indicated. Proteins with amino acid sequence homology, generally ~35%, have the same shade coding: 19.4K and 31.6K are unique to HAdV-F.

HAdVs represents a large family of genetically diverse pathogens. To date, more than 100 different HAdVs have been identified and classified into seven species, A to G (http://hadvwg.gmu.edu/ (accessed on 22 November 2019)). HAdVs cause partially overlapping, species-specific diseases associated with infections of the respiratory (species B, C, and E), urinary (species B), gastrointestinal (species A and F), and ocular (species D) systems [1]. HAdVs are highly contagious and can cause severe local outbreaks. Although healthy adults can generally control the virus, HAdV infections in children and immunocompromised individuals can be fatal [2,3,4,5]. HAdV devotes a considerable portion of its genome to modulation of host immune functions, which presumably enables some species to establish and maintain lifelong asymptomatic infections. The vast majority of HAdV genes involved in the modulation of host immune functions are grouped in the E3 region [6,7]. The E3 region is not essential for HAdV replication in cultured cells, but the fact that this transcription unit is always maintained in natural isolates strongly suggests that E3 gene products are critical for natural infections in humans [6,7]. Notably, E3 is one of the most divergent gene regions between species (see Figure 1). This genetic variability is not well understood, but it strongly suggests that E3 proteins play a role in the manifestation of species-specific tissue tropism and diseases [6]. The 19K protein of HAdV-C binds to and retains MHC class I molecules in the endoplasmic reticulum, thereby rendering HAdV-C-infected cells less efficient at presenting viral antigens and less sensitive to lysis by CD8+ T cells [8,9,10,11,12,13,14,15,16,17,18,19,20,21,22]. That this 19K gene is maintained in HAdV-B, -D, and -E (see Figure 1) underscores the critical need for these species to retain a MHC I-binding function. The x-ray crystal structures of AdV-C2 and AdV-E4 E3-19K bound to HLA-A2 provided insights into the mechanism of immune modulation [23,24]. Other E3 proteins were shown to function by inhibiting tumor necrosis factor activities and interfering with apoptotic cell death processes and leukocyte activation, ultimately suppressing the lysis of infected cells by NK cells [25] and references therein.

The enteric HAdV-F, which comprises HAdV-F40 and HAdV-F41, shows a narrow tropism for epithelial cells of the GI tract and is a leading etiologic agent of acute gastroenteritis in infants and young children worldwide [26,27,28,29]. Notably, there are marked differences in the E3 region of HAdV-F compared to the other species (see Figure 1): HAdV-F lacks the common E3-19K gene and has two genes, 19.4K and 31.6K, that are unique to that species [30,31,32]. To date, E3-19.4K and E3-31.6K proteins have not been characterized and we have no knowledge of their functions. The study of HAdV-F thus offers a unique opportunity to study how interactions between E3 proteins and the immune microenvironment at the site of infection contributes to viral tropism and pathogenicity. We suggest that the selective pressure on HAdV-F40 and HAdV-F41 by the immune system of the GI tract has led these viruses to adapt in order to replicate effectively in gut cells, and that E3-19.4K and/or E3-31.6K proteins play an important role in this process. More specifically, we suggest that HAdVs-F have evolved a function directed at suppressing the expression of MIC A and MIC B molecules on infected cells. MIC A and MIC B are stress-inducible surface ligands that are recognized by the NKG2D activating receptor expressed on NK cells to eliminate stressed cells. Given that the distribution of MIC activating ligands is largely restricted to intestinal epithelial cells under normal conditions, and that HAdVs-F are exquisitely adapted to replicate in the intestinal epithelium [33] and references therein, it might not be surprising that these viruses interfere with MIC A and MIC B to suppress immune surveillance by NK cells.

To advance our understanding of HAdVs-F, and given the significance of these viruses as pathogens, we have initiated a study to examine the effects of HAdV-F infection on cell surface expression of MIC ligands. We have established an in vitro culture system based on infection of human intestinal HCT116 cells with HAdVs-F from which we show that HAdV-F41 causes the intracellular sequestration of MIC B. These preliminary results support the hypothesis that interferences with NKG2D MIC ligands is a mechanism used by HAdVs-F to evade immune surveillance in the gut and may be a determinant of viral tropism.

## 2. Materials and Methods

### 2.1. Virus Growth and Cells

HAdV-F41 (ATCC^®^ VR-930™) was grown in 50–60% confluent HEK-293 cells (ATCC^®^ CRL-1573™) in DMEM (ATCC^®^ 30-2002) supplemented with 1–2% FBS (ATCC^®^ 30-2020™). Infection was done with virus at passage five at an MOI = 1. After infection, when cells show clear cytopathic effect (round up with increased nucleus size), cultures were harvested with a cell scraper and transferred to falcon tubes. Cell suspensions were centrifuged at 700× *g*, 4 °C for 10 min, and cells were resuspended in culture medium discharging the supernatant. Samples were subjected to three freeze/thaw cycles (−80 °C and 37 °C), then centrifuged at 1500× *g*, 4 °C for 10 min. Supernatants were aliquoted in small volumes and kept at −80 °C until use. To determine viral titers, an aliquot of the virus preparation was used for titration in HEK-293 cells via immunohistochemistry using the QuickTiter™ Adenovirus Quantitation Kit (Cell Biolabs, Catalog no. VPK-109, San Diego, CA, USA), following instructions by the manufacturer. Two cell types were used for the in vitro infection models: human colorectal carcinoma HCT116 cells (ATCC^®^ CCL-247, Manassas, VI, USA). HCT116 cells were grown in McCoy’s 5A medium (ATCC^®^ 30-2007™, Manassas, VI, USA) supplemented with 10% FBS.

### 2.2. Immunofluorescence Staining

For immunofluorescence (IF) staining, cells were grown on sterile glass coverslips placed on 12-well plates prior to infection with HAdV-F41 (MOI 0.5). After 2 days, cells were fixed in 4% PFA for 10 min, permeabilized with 0.1% triton x-100 for 20 min, and blocked with 1% PBS/BSA for 30 min. For virus staining, a rabbit anti-pVIII polyclonal Ab (provided by Dr. W. Wold, St-Louis University, St. Louis, MO, USA) was used. Cells were washed and stained for 1 h with a mixture of donkey anti-rabbit secondary Ab conjugated with rhodamine (Invitrogen, Catalog no. 31685, Waltham, MA, USA), and Phalloidin-iFluor 488 (Abcam, Catalog no. ab176753, Cambridge, UK) to stain actin fibers. MIC A and MIC B staining were done using primary mouse anti-MIC A and mouse anti-MIC B Abs. A goat anti-mouse-FITC was used as the secondary Ab. Coverslips were mounted on slides using ProLong™ Diamond Antifade with DAPI (Invitrogen, Catalog no. P36962, Waltham, MA, USA) and cured at 4 °C for 24 h in the dark. Samples were analyzed under an Olympus BX51 IF microscope coupled with a CCD camera to acquire individual channels of DAPI, alexa fluor 488 or rhodamine. Acquired channels were merged using ImageJ software v1.53a. Uninfected cells, and secondary Abs alone, produced no relevant signals in the rhodamine channel.

### 2.3. Flow Cytometry

HCT116 cells were infected with HAdV-F41 (MOI 0.5) and expression levels of MIC A and MIC B were determined on the cell surface and intracellularly by flow cytometry on days 2 and 4 post-infection. Infection was assessed based on the expression of intracellular hexon protein. At the harvest time, cells were scraped, washed in PBS by centrifugation at 700× *g* for 10 min, incubated with Zombie Violet Fixable Viability Kit (Biolegend, Catalog no. 423114, San Diego, CA, USA) at 1:500 for 30 min in the dark for discriminating live versus dead cells, washed, and fixed in 4% PFA for 20 min on ice. Cells were then washed and incubated with a mixture of anti-MIC A-phycoerythrin (PE) (Sino Biological Catalog no. 12302-MM04-P, Beijing, China) and anti-MIC B-allophycocyanin (APC) (Sino Biological Catalog no. 10759-MM12-A, Beijing, China) Abs for 40 min on ice. Isotype Abs recommended by the manufacturer, as well as uninfected HCT116 cells, were used as negative controls. In the case of samples prepared for extra- and intra-cellular staining, cells were incubated with Ab cocktail for surface staining prior to permeabilization with 0.1% triton x-100 for 10 min at RT. Hexon staining was carried out using a 2Hx-2 monoclonal anti-hexon Ab (provided by Dr. W. Wold, St-Louis University, St. Louis, MO, USA) [34] with further detection using a secondary anti-mouse-FITC Ab. After staining, cells were washed 2 times in PBS, resuspended in 300 μL PBS, and data were acquired on a Gallios flow instrument (Beckman & Coulter, Brea, CA, USA). Samples were analyzed offline using FlowJo software v10 considering only live cells, after exclusion of cell debris and aggregates.

### 2.4. Protein Sequence Analysis

The GenBank accession number for HAdV-F41 E3 region is M85254 [32]. Bioinformatics software and servers used for protein sequence analysis were: SignalP v. 5.0 (DTU Bioinformatics, Denmark) [35], SMART (EMBL, Heidelberg, Germany) [36], TMHMM v. 2.0 (DTU Bioinformatics, Denmark) [37], and PHYRE v. 2.0 (Imperial College, London, UK) [38].

## 3. Results

### 3.1. E3 Region

E3 is one of the most divergent gene regions between species (Figure 1): while some E3 proteins are found in all species, others have counterparts in only a few species, and some E3 proteins are unique to a given species. Remarkably, HAdV-F lacks the common E3-19K protein and instead expresses two proteins, 19.4K and 31.6K, that are unique to this species (Figure 1). To date, the functions of these proteins are unknown.

### 3.2. In Vitro Models of HAdV-F Infection

**Figure 2:**
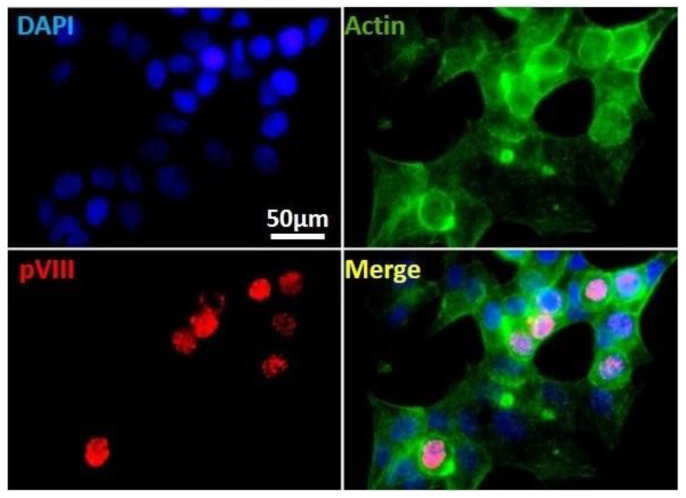
Immunofluorescence staining of HAdV pVIII protein in HAdV-F41-infected HCT116 cells. Cells infected with HAdV-F41 (MOI 0.5) at day 2 post-infection showing nuclear localization of the viral structural pVIII protein (red). Actin fibers and cell chromatin are presented in green and blue, respectively. Samples were analyzed under an Olympus BX51 IF microscope coupled with a CCD camera Acquired channels were merged using ImageJ software v1.53a. Uninfected cells or secondary Ab alone yielded no relevant signals.

That HAdVs-F are one of the least characterized species may be due to the difficulty of propagating these viruses in most common human cell culture systems that permit replication of all other HAdVs. We established an in vitro culture system for infection of human intestinal HCT116 with HAdV-F41 (Figure 2). To characterize this cell system, we used IF staining of HAdV structural proteins hexon and pVIII as a way to track infected cells. HCT116 cells were infected with HAdV-F41 (MOI 0.5) and results shows a clear nuclear staining of pVIII on day 2 post-infection, consistent with the permissiveness of these cells for HAdV-F41 infection. The results establish new conditions for HAdV-F41 infection in HCT116 cells.

### 3.3. HAdV-F41 Interferes with Cell Surface Expression of MIC B

**Figure 3:**
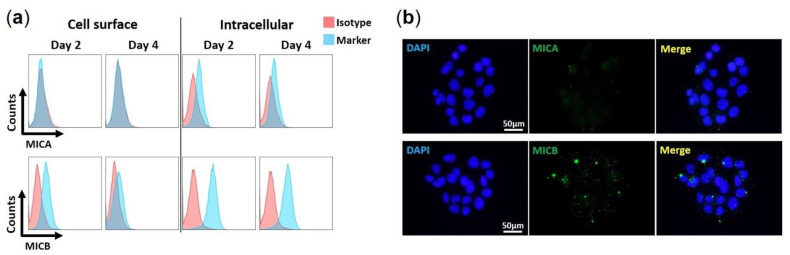
Expression of MIC ligands in uninfected HCT116 cells. (**a**) Flow cytometry histograms showing levels of MIC A and MIC B on the surface and in the intracellular environment of uninfected HCT116 cells. Cells were harvested at day 2 and 4 in culture. Isotype Abs recommended by the manufacturer were used as negative controls. Sample were analyzed on a Gallios (Beckman & Coulter, Brea, CA, USA) flow instrument and analysis was done offline using FlowJo software v10. Histograms are gated on live cells, after exclusion of cell debris and aggregates. (**b**) Immunofluorescence staining of MIC A and MIC B in uninfected HCT116 cells at day 2 in culture, with DAPI in blue and MICs in green. Data are representative of three independent experiments.

We examined if HAdV-F41 impairs the cell surface expression of MIC A and MIC B in HCT116 cells by flow cytometry and IF. We first characterized the basal expression levels of MIC ligands in uninfected HCT116 cells over four days. Results show that for both MIC A and MIC B, expression levels are higher intracellularly than on the cell surface (Figure 3a). Furthermore, MIC B is more abundant overall than MIC A (Figure 3a,b), and MIC A is negligibly expressed on HCT116 cells (Figure 3a). Finally, it is important to note that, in uninfected HCT116 cells, MIC B cell surface expression levels decreased slightly from day 2 to day 4 (Figure 3a). This may be due to the proteolytic shedding of MIC B from the cell surface, a process that occurs during normal cell growth and the expression of MIC proteins [39].

**Figure 4:**
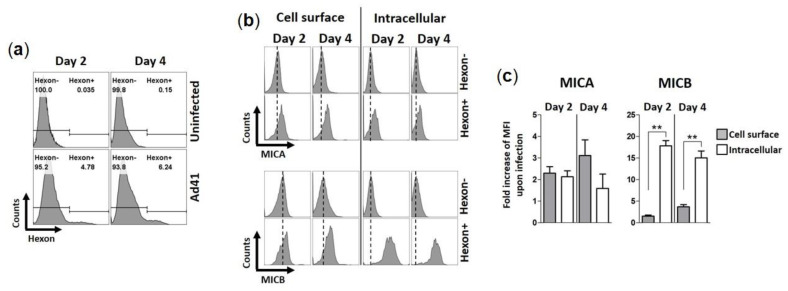
Expression of MIC ligands in HAdV-F41-infected HCT116 cells. HCT116 cells were infected with HAdV-F41 (MOI 0.5) and levels of MIC A and MIC B were assessed on the cell surface and intracellularly by flow cytometry, on day 2 and 4 post-infection. (**a**) Flow cytometry histograms showing staining of HAd41-infected cells (Hexon^+^) using a 2Hx-2 monoclonal anti-hexon Ab [34] with further detection with a secondary anti-mouse-FITC Ab. (**b**) Flow cytometry histogram showing expression levels of MIC A and MIC B on the cell surface and intracellularly. Hexon^-^ populations were gated from uninfected samples. Dashed lines represent the MFI levels of MIC A or MIC B on uninfected samples. (**c**) Fold increases in expression of MIC A and MIC B upon HAdV-F41 infection were calculated as MFI^hexon+^ / MFI^hexon-^. ** *p* < 0.01 defined by t student test.

**Figure 5:**
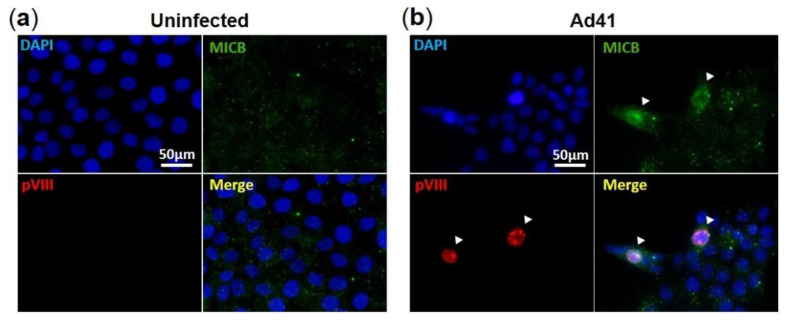
Immunofluorescence assay of MIC B in HAdV-F41-infected HCT116 cells. IF assay showing (**a**) uninfected and (**b**) HAdV-F41-infected HCT116 cells (MOI 0.5; day 2). HAdV-F41 was traced using a rabbit polyclonal anti-pVIII Ab and a secondary goat anti-rabbit-Rhodamine. MIC B detection was performed using a mouse anti-MIC B Ab followed by incubation with a goat anti-mouse-FITC. Cell nuclei were counterstained with DAPI. Arrows show HAdV-F41-infected cells exhibiting stronger signals of MIC B compared to uninfected cells.

We then examined if HAdV-F41 modulates the expression levels of MIC ligands in infected HCT116 cells over four days using flow cytometry. To account for the natural proteolytic shedding of MICs, the controls consisted of uninfected HCT116 cells collected at each time point. HAdV-F41-infected cells were detected using a monoclonal anti-hexon Ab (Figure 4a). Results show that MIC A expression levels, whether on the cell surface or intracellularly, are consistently higher in hexon^+^ cells than hexon^-^ cells (Figure 4b). The same observation was made for MIC B (Figure 4b). Thus, the expression of MIC ligands is upregulated by HCT116 cells infected with HAdV-F41. The data in Figure 4b were further analyzed by considering changes in median fluorescence intensity (MFI) of MIC expression levels in hexon^+^ cells relative to hexon^-^ cells and the values plotted as “fold increase” (Figure 4c, see legend). The analysis revealed that hexon^+^ cells expressed significantly more MIC B in intracellular compartments relative to hexon^-^ cells, 18-fold increase on day 2 versus 15-fold increase on day 4. In contrast, there was only a small increase of MIC B expression on the cell surface, 1.5-fold on day 2 versus 3.7-fold on day 4 (Figure 4c). Thus, although HAdV-F41 cause an upregulation of MIC B in HCT116 cells, this did not lead to increased expression of the ligand on the cell surface suggesting that MIC B is largely sequestered intracellularly in infected cells. A similar trend for MIC A could not be observed in these cells, instead similar relative changes in intracellular and cell surface expression levels of MIC A were determined (Figure 4c). Finally, consistent with the flow cytometry results showing higher levels of MIC B expression in hexon^+^ cells (Figure 4b), IF analysis showed that fluorescence intensity signals are stronger in HAdV-F41-infected cells (shown as pVIII^+^ cells) (Figure 5b) relative to uninfected cells (pVIII^-^ cells) (Figure 5a).

### 3.4. E3-19.4K and E3-31.6K Proteins

Given the uniqueness of E3-19.4K and E3-31.6K to HAdV-F, and the strong possibility that these proteins participate in immune evasion functions in the gut, we characterized their basic properties. HAdV-F41 19.4K has 173 residues (Supplementary Figure S1) and its amino acid sequence is 99% identical to its counterpart in HAdV-F40 except for a single amino acid change at residue 144, which is an isoleucine in HAdV-F41 and asparagine in HAdV-F40 [31,32]. An analysis of HAdV-F41 19.4K sequence using the Bioinformatics software SignalP-5.0, SMART, and TMHMM [35,36,37,38] predicts that the protein is a type I transmembrane protein with the signal sequence comprising the first 15 or 18 N-terminal residues (depending on the software used) and transmembrane domain spanning residues 144 to 166 (Supplementary Figure S1). Because residue 144 is predicted to be membrane-localized, the ectodomains of E3-19.4K are expected to be identical between HAdV-F40 and HAdV-F41. Secondary structure predictions using the Phyre2 package indicated that E3-19.4K has 13% α-helix, 58% β-strand, and 20% disordered regions, with the putative transmembrane domain correctly identified as an α-helix. The accuracy of these predictions awaits a determination of the three-dimensional structure of E3-19.4K. A BLAST analysis indicated that the amino acid sequence of HAdV-F41 E3-19.4K shows no homology to the common E3-19K immunomodulatory proteins of species B, C, D, and E. The BLAST analysis also revealed that HAdV-F41 E3-19.4K is 50% homologous to the E3 CR1-α1 protein of HAdV-G52 [40]. The homology comprises residues 39 to 122 (underlined in Supplementary Figure S1) and represents a conserved region within E3 named “adenoE3CR1rpt” (also known as 6.7K) [41,42]. HAdV-G52 has not been extensively studied and the function of its CR1-α1 protein is unknown. However, it was shown that HAdV-C2 CR1-α1 directs E3-19K to the ER [43] and can also cooperate with the RID proteins to evade TNF-α-induced NK-κB activation [44]. The lack of general information about E3 CR1-α1 proteins makes it difficult to draw any conclusions on the role of this motif in E3-19.4K.

HAdV-F41 E3-31.6K has 276 residues (Supplementary Figure S2) and its amino acid sequence is 99% homologous to that of HAdV-F41 E3-31.6K, except for two amino acid changes at residues 192 and 264 which are both glutamic acid in Ad41 and lysine in Ad40 [31,32]. E3-31.6K is predicted to be a type I transmembrane protein with the signal sequence comprising the first 16 N-terminal residues and the transmembrane domain spanning residues 234 to 256 (Supplementary Figure S2). Secondary structure predictions using Phyre^2^ indicated that E3-31.6K has 17% α-helix, 50% β-strand, and 11% disordered regions, with the putative transmembrane domain correctly identified as an α-helix. A BLAST analysis indicated that the amino acid sequence of HAdV-F40 E3-31.6K shows no homology to E3-19K proteins. No other significant sequence homology was identified.

## 4. Discussion

HAdVs-F replicate preferentially in the GI tract and cause severe gastroenteritis in children. The selective pressure on HAdVs-F by the microenvironment of the gut has led these viruses to eliminate the E3-19K gene, and presumably the MHC I-binding function, and to evolve two new E3 genes that are conspicuously absent in all other species. This raises intriguing questions on the underlying mechanisms by which HAdVs-F are exquisitely adapted to the GI tract. Given that E3 proteins modulate various host immune functions [6,7], it is reasonable to assume that the distinct pathogenicity of HAdVs-F stems in some way from interactions of their E3 proteins with the immune system of the gut. On that basis, we have investigated the effect of HAdV-F infection on stress-induced MIC A and B molecules. MIC A and MIC B are normally expressed at low levels almost exclusively on intestinal epithelial cells and engage with activating receptors on NK cells as part of host immunosurveillance of stressed cells.

HAdVs-F are notoriously difficult to grow in most cell culture systems [45,46,47] and whether this characteristic arises from a feature unique to this species, namely that they contain two different fiber proteins (long and short) and unique penton base proteins, is unclear [48,49,50]. We have developed optimal cell culture conditions for infection of intestinal HCT 116 cells with HAdV-F41. Our results showed that HAdV-F41 infection of HCT116 cells upregulated the expression of MIC A and MIC B relative to uninfected cells, on the cell surface as well as intracellularly. These results are consistent with the role of MIC A and MIC B as stress-inducible ligands and underline a possible role for the NKG2D pathway in HAdV-F infection. Our results also showed that for MIC B, this response did not however lead to a significant increase of the ligand on the cell surface. Instead, MIC B was largely sequestered intracellularly. Thus, although HAdV-F41 infection upregulates the expression levels of MIC B in HCT116 cells, the ligand remained inside infected cells. A similar trend for MIC A could not be observed in HCT116 cells and therefore it remains to be further evaluated if HAdVs-F selectively target MIC B-the selective targeting of MIC ligands (MIC A or MIC B) has been reported previously for several human viruses [51,52,53,54,55]. Taken together, we showed for the first time that HAdV-F41 infection of HCT116 cells led to the intracellular sequestration of the NKG2D activating ligand MIC B. Whether our findings represent a viral escape mechanism to prevent recognition and elimination of HAdV-F41-infected cells in the gut by NK cells requires further investigation.

Our results raise important questions concerning the mechanism by which HAdV-F sequesters MIC B inside cells, and the viral factor responsible for this effect. The E3-19.4K and E3-31.6K proteins are highly conserved in HAdVs-F, 99% amino acid sequence identity between HAdV-F40 and HAdV-F41, which suggests that these proteins are critical for viral tropism or virulence in the gut. Interestingly, it was shown previously that infection of human fibroblasts by HAdV-C2 and HAdV-C5 led to the sequestration of MIC A and MIC B inside infected cells [56], an effect that was attributed to the E3-19K protein [56,57]. HAdVs-C are tropic for epithelial cells of the lungs and cause respiratory illnesses. However, these viruses can also cause GI symptoms, as part of a systemic infection with accompanying respiratory disorders, and are persistently detected in stools of healthy and infected individuals. Furthermore, tumorigenic HAdV-A12 of species A, which is also associated with gastroenteritis, was shown to suppress the expression of NKG2D activating ligands on transformed mouse and rat cells through the transcriptional repression of these ligands [58]. The protein responsible for this effect has not yet been identified. Taken together, interferences with NKG2D activating ligands may be an important mechanism by which HAdVs mediate immune evasion in the GI tract, most especially for species F. In this context, others have shown, using a model system of human enteroids, that HAdV-F41 was resistant to the activity of enteric alpha-defensin 5 while in contrast HAdV-C5 was neutralized [59,60]. These results show that a host factor such as alpha-defensin 5, an innate defense peptide expressed in the crypts of the intestine, could also modulate the tropism of enteric species F.

In conclusion, we showed that HAdV-F41 sequesters MIC B inside infected cells. This represents a novel function of HAdVs-F. HAdVs-F have received considerably less attention than the other HAdVs, despite being significant pathogens, and our findings cast a new light on how these viruses, under immune pressure in the GI tract, have remarkably adapted to this site. Future investigations will provide more details about this function and the viral protein responsible for it, and will reveal if the suppression of MIC ligands impairs the recognition of HAdV-F-infected cells by NK cells.

## Data Availability

Not applicable.

## Supplementary Materials

The following are available online at https://www.mdpi.com/article/10.3390/v13071289/s1, Figure S1: The amino acid sequence of HAdV-F41 E3-19.4K protein (residues 1-173), Figure S2: The amino acid sequences of HAdV-F41 E3-31.6K protein (residues 1- 276).

## References

[1] Berk A., Fields B.N., Knipe D.M., Howley P.M. (2013). Adenoviridae. Fields Virology.

[2] Lion T. (2014). Adenovirus infections in immunocompromised patients. Clin. Microbiol. Rev..

[3] Hoffman J.A. (2009). Adenovirus infections in solid organ transplant recipients. Curr. Opin. Organ. Transplant..

[4] Feghoul L., Chevret S., Cuinet A., Dalle J.H., Ouachee M., Yacouben K., Fahd M., Guerin-El Khourouj V., Roupret-Serzec J., Sterkers G. (2015). Adenovirus infection and disease in paediatric haematopoietic stem cell transplant patients: Clues for antiviral preemptive treatment. Clin. Microbiol. Infect..

[5] Fisher B.T., Boge C.L.K., Petersen H., Seif A.E., Bryan M., Hodinka R.L., Cardenas A.M., Purdy D.R., Loudon B., Kajon A.E. (2018). Outcomes of human adenovirus infection and disease in a retrospective cohort of pediatric hematopoietic cell transplant patients. J. Pediatr. Infect. Dis. Soc..

[6] Burgert H., Blusch J.H. (2000). Immunomodulatory functions encoded by the E3 transcription unit of adenoviruses. Virus Genes.

[7] Wold W.S.M., Gooding L.R. (1991). Region E3 of adenovirus: A cassette of genes involved in host immunosurveillance and virus-cell interactions. Virology.

[8] Burgert H.-G., Kvist S. (1987). The E3/19K protein of adenovirus type 2 binds to the domains of histocompatibility antigens required for CTL recognition. EMBO J..

[9] Burgert H.-G., Kvist S. (1985). An adenovirus type 2 glycoprotein blocks cell surface expression of human histocompatibility class I antigens. Cell.

[10] Kvist S., Ostberg L., Persson H., Philipson L., Peterson P.A. (1978). Molecular association between transplantation antigens and cell surface antigen in adenovirus-transformed cell line. Proc. Natl. Acad. Sci. USA.

[11] Paabo S., Bhat B.M., Wold W.S.M., Peterson P.A. (1987). A short sequence in the COOH terminus makes an adenovirus membrane glycoprotein a resident of the endoplasmic reticulum. Cell.

[12] Sester M., Ruszics Z., Mackley E., Burgert H.-G. (2013). The transmembrane domain of the adenovirus E3/19K protein acts as an endoplasmic reticulum retention signal and contribute to intracellular sequestration of major histocompatibility complex class I molecules. J. Virol..

[13] Cox J.H., Bennink J.R., Yewdell J.W. (1991). Retention of adenovirus E19 glycoprotein in the endoplasmic reticulum is essential to its ability to block antigen presentation. J. Exp. Med..

[14] Liu H., Stafford W.F., Bouvier M. (2005). The endoplasmic reticulum lumenal domain of the adenovirus type 2 E3-19K binds to peptide-filled and peptide-deficient HLA-A*1101 molecules. J. Virol..

[15] Liu H., Fu J., Bouvier M. (2007). Allele- and locus-specific recognition of class I MHC molecules by the immunomodulatory E3-19K protein from adenovirus. J. Immunol..

[16] Fu J., Li L., Bouvier M. (2011). Adenovirus E3-19K proteins of different serotypes and subgroups have similar, yet distinct, immunomodulatory functions towards major histocompatibility class I molecules. J. Biol. Chem..

[17] Fu J., Bouvier M. (2011). Determinants of the endoplasmic reticulum (ER) lumenal-domain of the Adenovirus serotype 2 E3-19K protein for association with and ER-retention of major histocompatibility complex class I molecules. Mol. Immunol..

[18] Flomenberg P., Piaskowski V., Truitt R.L., Casper J.T. (1996). Human adenovirus-specific CD8+ T-cell responses are not inhibited by E3-19K in the presence of gamma interferon. J. Virol..

[19] Andersson M., McMichael A., Peterson P.A. (1987). Reduced allorecognition of adenovirus-2 infected cells. J. Immunol..

[20] Burgert H.-G., Maryanski J.L., Kvist S. (1987). “E3/19K” protein of adenovirus type 2 inhibits lysis of cytolytic T lymphocytes by blocking cell surface expression of histocompatibility class I antigens. Proc. Natl. Acad. Sci. USA.

[21] Tanaka Y., Tevethia S.S. (1988). Differential effect of adenovirus 2 E3/19K glycoprotein on the expression of H-2Kb and H-2Db class I antigens and H-2Kb- and H-2Db-restricted SV40-specific CTL-mediated lysis. Virology.

[22] Rawle F.C., Tollefson A.E., Wold W.S.M., Gooding L.R. (1989). Mouse anti-adenovirus cytoxic T lymphocytes. J. Immunol..

[23] Li L., Muzahim Y., Bouvier M. (2012). Crystal structure of adenovirus E3-19K bound to HLA-A2 reveals mechanism for immunomodulation. Nat. Struc. Mol. Biol..

[24] Li L., Santarserio B.D., Bouvier M. (2016). Structure of the Adenovirus Type 4 (Species E) E3-19K/HLA-A2 complex reveals species-specific features in MHC I recognition. J. Immunol..

[25] Oliveira E.R.A., Bouvier M. (2019). Immune evasion by adenoviruses: A window into host-virus adaptation. FEBS Lett..

[26] Lee J.I., Lee G.-C., Chung J.Y., Han T.H., Lee Y.K., Kim M.S., Lee C.H. (2012). Detection and molecular characterization of adenoviruses in Korean children hospitalized with acute gastroenteretitis. Microbiol. Immunol..

[27] LaRosa G., Libera S.D., Petricca S., Iaconelli M., Donia D., Saccucci P., Cenko F., Xhelilaj G., Divizia M. (2015). Genetic diversity of human adenovírus in children with acute gastreoenteretitis, Albania, 2013–2015. Biomed. Res. Intern..

[28] Afrad M.H., Avzun T., Haque J., Haguw W., Hossain M.E., Rahman A.F.M.R., Ahmed S., Faruque A.S.G., Rahman M.Z., Rahman M. (2017). Detection of enteric- and non-enteric adenoviruses in gastreoenteretitis patients, Bangladesh, 2012–2015. J. Med. Virol..

[29] Kumthip K., Khamrin P., Ushijima H., Maneekarn N. (2019). Enteric- and non-enteric adenoviruses associated with acute gastreoenteretitis in pediatric patients in Thailand, 2011 to 2017. PLoS ONE.

[30] Davison A.J., Telford A.R., Watson M.S., McBride K., Mautner V. (1993). The DNA sequence of adenovirus type 40. J. Mol. Biol..

[31] Bailey A., Mautner V. (1994). Phylogenetic relationships among adenovirus serotypes. Virology.

[32] Yeh H.-Y., Pieniazek N., Pieniazek D., Luftig R. (1996). Genetic organization, size, and complete sequence of early region 3 genes of human adenovirus type 41. J. Virol..

[33] Kosulin K. (2019). Intestinal HAdV infection: Tissue specificity, persistence, and implications for antiviral therapy. Viruses.

[34] Cepko C.L., Whetsone C.A., Sharp P.A. (1983). Adenovirus hexon monoclonal antibody that is group specific and potentially useful as a diagnostic reagent. J. Clin. Microbiol..

[35] Armenteros J.J.A., Tsirigos K.D., Sønderby C.K., Petersen T.N., Winther O., Brunak S., von Heijne G., Nielsen H. (2019). SignalP 5.0 improves signal peptide predictions using deep neural networks. Nat. Biotech..

[36] Letunic I., Bork P. (2020). SMART: Recent updates, new developments and status in 2020. Nucleic Acid Res..

[37] Krogh A., Larsson B., von Heijne G., Sonnhammer E.L.L. (2011). Predicting transmembrane protein topology with a hidden Markov model: Application to complete genomes. J. Mol. Biol..

[38] Kelley L., Jefferys B. (2011). Phyre2: Protein Homology/Analogy Recognition Engine V 2.0.

[39] Moncaya G., Lin D., McCarthy M.T., Watson A.A., O’Calaghan C.A. (2017). MICA expression is regulated by cell adhesion and contact in a FAK/Src-dependent manner. Front. Immunol..

[40] Jones M.S., Harrach B., Ganac R.D., Gozum M.M.A., de la Cruz W.P., Riedel B., Pan C., Delwart E.L., Schnurr D.P. (2007). New adenoviruses species found in a patient presenting with gastroenteritis. J. Virol..

[41] Davison A.J., Benko M., Harrach B. (2003). Genetic content and evolution of adenoviruses. J. Gen. Virol..

[42] Deryckere F., Burgert H.-G. (1996). Early region 3 adenovirus type 19 (subgroup D) encodes an HLA-binding protein distinct from that of subgroups B and C. J. Virol..

[43] Rawls-Wilson J., Deutscher S.L., Wold W.S.M. (1994). The signal-anchor domain of adenovirus E3-6.7K, a type III integral membrane protein can direct adenovirus E3-gp19K, a type I integral membrane protein, into the membrane of the endoplasmic reticulum. Virology.

[44] Benedict C.A., Norris P.S., Prigozy T.I., Bodmer J.-L., Mahr J.A., Garnett C.T., Martinnon F., Tschopp J., Gooding L.R., Ware C.F. (2001). Three adenovirus E3 proteins cooperate to evade apoptosis by tumor necrosis factor-related apoptosis-inducing ligand receptor-1 and -2. J. Biol. Chem..

[45] Tiemessen C.T., Kidd A.H. (1994). Adenoviruses type 40 and 41 growth in vitro: Host range diversity reflected by differences in patterns of DNA replication. J. Virol..

[46] de Jong J.C. (1983). Candidate adenoviruses 40 and 41: Fastidious adenoviruses from human infant stool. J. Med. Virol..

[47] Witt D.J., Bousquet E.B. (1988). Comparison of enteric adenovirus infection in various human cell lines. J. Virol. Methods.

[48] Kidd A.H., Chroboczek J., Ruigrok R.W. (1983). Adenovirus type 40 virions contain two distinct fibers. Virology.

[49] Yeh H.Y., Pieniazek N., Pieniazek D., Gelderblom H., Luftig R.B. (1994). Human adenovirus type 41 contains two fibers. Virus Res..

[50] Albinsson B., Kidd A.H. (1999). Adenovirus type 41 lacks an RGD alpha(v)-integrin binding motif on the penton base and undergoes delayed uptake in A549 cells. Virus Res..

[51] Jonjic S., Babic M., Polic B., Krmpotic A. (2008). Immune evasion of natural killer cells by viruses. Curr. Opin. Immunol..

[52] Cosman D., Müllberg J., Sutherland C.L., Chin W., Armitage R., Fanslow W., Kubin M., Chalupny N.J. (2001). ULPBs, novel MHC class I-related molecules, bind to CMV glycoprotein UL16 and stimulates NK cytotoxicity through NKG2D receptor. Immunity.

[53] Ashiru O., Bennett N.J., Boyle L.H., Thomas M., Trowsdale J., Wills M.R. (2009). NKG2D ligand MICA is retained in the cis-Golgi apparatus by human cytomegalovirus protein UL142. J. Virol..

[54] Schneider C.L., Hudson A.W. (2011). The human herpesvirus-7 (HHV-7) U21 immunoevasin subverts NK-mediated cytotoxicity through modulation of MICA and MICB. PLoS Pathog..

[55] Thomas M., Boname J.M., Field S., Nejentsev S., Salio M., Cerundolo V., Wills M., Lehner P.J. (2008). Down-regulation of NKG2D and NKp80 ligands by Kaposi’s sarcoma-associated herpesvirus K5 protects against NK cell cytotoxicity. Proc. Natl. Acad. Sci. USA.

[56] McSharry B.P., Burgert H.G., Owen D.P., Stanton R.J., Prod’homme V., Sester M., Koebernick K., Groh V., Spies T., Cox S. (2008). Adenovirus E3/19K promotes evasion of NK cell recognition by intracellular sequestration of the NKG2D ligands major histocompatibility complex class I chain-related proteins A and B. J. Virol..

[57] Sester M., Ruszics Z., Mackley E., Burget H.-G. (2010). Conserved amino acids within the adenovirus 2 E3/19K protein differentially affect downregulation of MHC class I and MICA/B proteins. J. Immunol..

[58] Heyward C.Y., Patel R., Mace E.M., Grier J.T., Guan H., Makrygiannis A.P., Orange J.S., Ricciardi R.P. (2012). Tumorigenic adenovirus 12 cells evade NK cell lysis by reducing the expression of NKG2D ligands. Immunol. Lett..

[59] Smith J.G., Silvestry M., Lindert S., Lu W., Nemerow G.R., Stewart P.L. (2010). Insights into the mechanisms of adenovirus capsid disassembly from studies of defensin neutralization. PLoS Pathog..

[60] Holly M.K., Smith J.G. (2018). Adenovirus infections of human enteroids reveals interferon sensitivity and preferential infection of globlet cells. J. Virol..

