# Supplementary material for: Intracellular Sequestration of the NKG2D Ligand MIC B by Species F Adenovirus
Source: Viruses. 2021 Jul 1;13(7):1289. doi: 10.3390/v13071289 (PMC8310058; doi:10.3390/v13071289)
Supplement: Supplementary file 1 [file viruses-13-01289-s001.zip › viruses-1241645-supplementary.pdf]

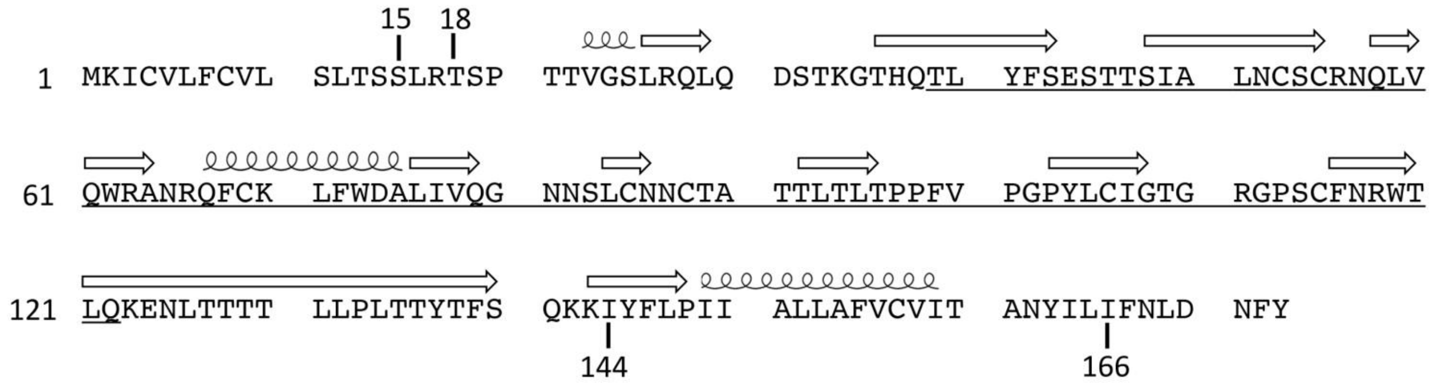

Figure S1. The amino acid sequence of HAdV-F41 E3-19.4K protein (residues 1–173). The predicted signal peptide, residues 1 to 15 or 1 to 18 (depending on the prediction software used), and transmembrane domain, residues 144 to 166, are indicated. The conserved adenoE3CR1rpt motif (residues 39–122) is underlined. Secondary structure predictions are indicated at the top of the sequence.

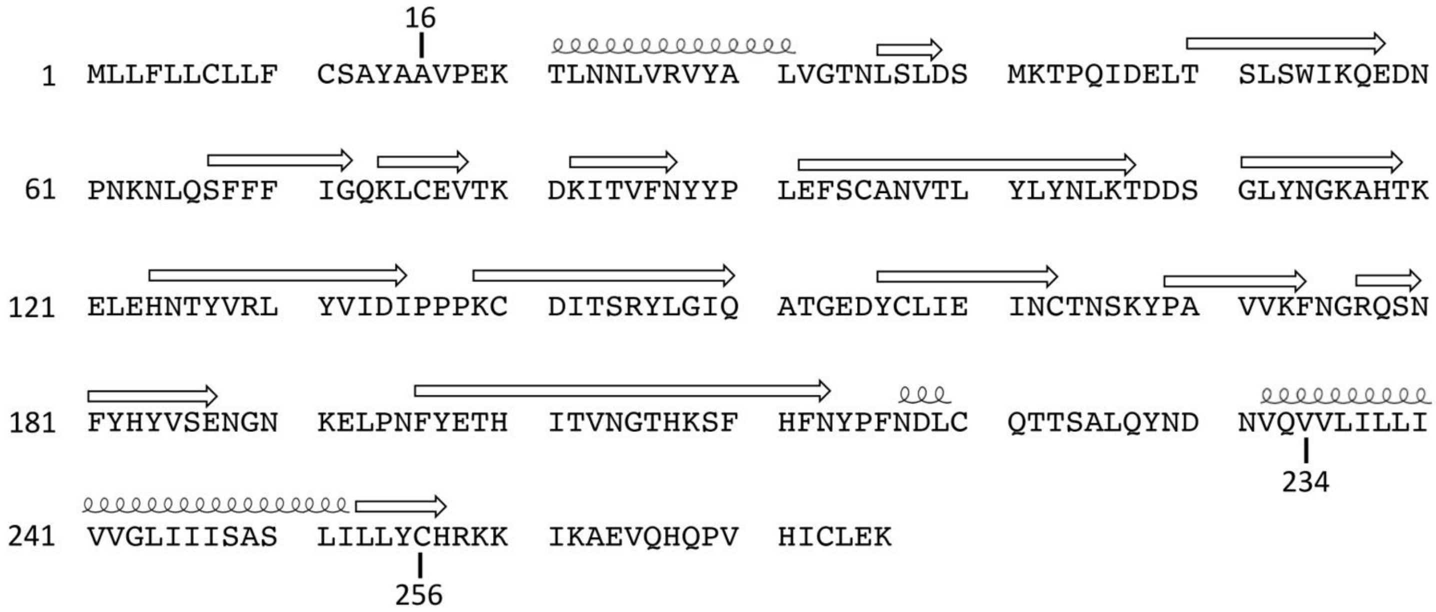

Figure S2. The amino acid sequences of HAdV-F41 E3-31.6K protein (residues 1–276). The predicted signal peptide, residues 1 to 16, and transmembrane domain, residue 234 to 256, are indicated. Secondary structure predictions are indicated at the top of the sequence.
